# TMT-Based Quantitative Proteomic Analysis Identification of Integrin Alpha 3 and Integrin Alpha 5 as Novel Biomarkers in Pathogenesis of Acute Aortic Dissection

**DOI:** 10.1155/2020/1068402

**Published:** 2020-07-02

**Authors:** Lingyu Xing, Yuan Xue, Yilin Yang, Ping Wu, Catherine C. L. Wong, Haojun Wang, Zhenju Song, Dongwei Shi, Chaoyang Tong, Chenling Yao, Guorong Gu

**Affiliations:** ^1^Department of Emergency Medicine, Zhongshan Hospital, Fudan University, Shanghai 200032, China; ^2^National Centre for Protein Science Shanghai, Shanghai 200032, China; ^3^Centre for Precision Medicine Multi-Omics Research (CPMMR), Health Science Centre, Peking University, Beijing 102206, China

## Abstract

**Background:**

Acute aortic dissection (AAD) is a devastating cardiovascular disease with a high rate of disability and mortality. This disease often rapidly progresses to fatal multiple organ hypoperfusion, and the incidence has been increasing in recent years. However, the molecular mechanisms have yet to be clarified. This study is aimed at identifying the differential abundance proteins (DAPs) of aortic arch tissues in patients with AAD by proteomics and select possible proteins involved in AAD pathogenesis.

**Methods:**

The fresh aortic arch tissues obtained from 5 AAD patients and 1 healthy donor were analyzed by amine-reactive tandem mass tag (TMT) labelling and mass spectrometry; then, the pathological sections of another 10 healthy donors and 20 AAD patients were chosen to verify the proteomic results by immunohistochemistry.

**Results:**

Of 809 proteins identified by proteomic analysis, 132 differential abundance proteins (DAPs) were screened, of which 100 proteins were significantly downregulated while 32 upregulated. Among 100 downregulated proteins, two proteins with known function, integrin alpha 3 (ITGA-3) and ITGA-5, were selected as target proteins involved in AAD pathogenesis. Two target DAPs were verified by immunohistochemisty, and the results showed that the integrated option density (IOD) of ITGA-3 and ITGA-5 in AAD patients was significantly lower than that in healthy donors, which were consistent with the proteomic results (*P* < 0.001).

**Conclusion:**

ITGA-3 and ITGA-5 represent novel biomarkers for the pathogenesis of AAD and might be a therapeutic target in the future.

## 1. Introduction

Acute aortic dissection (AAD) is one of the most serious acute aorta syndrome characterized by severe chest and/or back pain. AAD of the ascending aorta is highly lethal (a mortality rate of 1%-2% per hour early after symptom onset) that requires prompt diagnosis and surgical intervention to optimize outcomes [1–3]. The etiologies include not only mechanical stimulation such as hypertension and trauma but also vascular injury caused by atherosclerosis, inflammation, and connective tissue disease [4]. Despite recent progress in recognition of the diagnostic and therapeutic advances, clinicians are far from comfortable in defining an optimal therapy to manage aortic dissection, and AAD is still a focus and key point in the field of cardiovascular surgery and critical care so far.

AAD is a surgical emergency occurring when an intima of the aorta is damaged and ruptured and blood enters the media creating a false lumen between the two layers. The typical pathophysiological changes include large modifications to the extracellular matrix, oxidative stress, inflammatory response, vascular smooth muscle (VSM) hypertrophy, and high expression of metalloproteinase, which directly lead to the compensatory hyperplasia of collagen fiber, apoptosis and phenotypic transformation of vascular smooth muscle cells (VSMCs), and steady degradation of the extracellular matrix proteins in the aortic wall [5–13]. Current research showed that the activation of the TGF-*β*1 signaling pathway and high expression of its downstream matrix metalloproteinase (MMPs) are involved in the pathogenesis of AAD [14–16]. Studies also have found that the PI3K/AKT signaling pathway contributes to phenotypic switching of vascular smooth muscle cell (VSMC) and induces apoptosis [17–19]. However, detailed molecular mechanisms still need to be explored.

**Figure 1:**
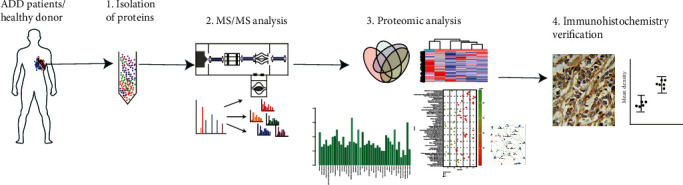
Experimental flowchart. The aortic arch tissues of 5 patients with AAD were extracted during surgery. The peptides labelled with TMT were prepared, and a MS/MS analysis was performed. The proteomic data were analyzed, and the DAPs were selected and verified by immunohistochemistry.

Quantitative mass spectrometry (MS) is an adjunctive tool to help understand the mechanisms of many diseases [20]. Large-scale identification of differential abundance proteins (DAPs) can be used to identify key proteins, and immunohistochemistry is an important traditional technique used to verify the selected proteins. The objective of this study was to identify and quantify DAPs of aortic arch tissues in patients with AAD using TMT-labelled MS and further to verify the selected DAPs of interest using immunohistochemistry, by which we hope to find some possible proteins involved in the pathogenesis of AAD. A flowchart to illustrate this study is shown in Figure 1.

## 2. Materials and Methods

### 2.1. Tandem Mass Tag Labelling

#### 2.1.1. Sample

The dissected aortic tissues (aortic arch including all layers of the wall: intima, media, and adventitia) from 5 AAD patients were extracted during surgery. In addition, one piece of healthy aortic arch tissue was obtained from a 44-year-old male donor. CTA was performed in all patients to confirm the diagnosis (all were Stanford type A dissection). Four out of them with hypertension history complained of chest and/or back pain, while one 22-year-old patient, with no history of hypertension and trauma, no family history of Marfan syndrome, and no typical clinical symptoms, was diagnosed with AAD. The clinical features and more detailed description of dissected aortic samples are summarized in Supplementary Table 1.

All tissues were collected as typical lesion specimens of approximately 1.0 × 1.0 cm^2^ in size. Specimens were immediately placed in an ice-bath, RNA-free specimen box and transferred to a −80°C refrigerator for preservation. The study was approved by the ethics committee of Zhongshan Hospital, Fudan University (Shanghai, China). Informed consent was obtained from patients or their legal surrogates before enrolment.

#### 2.1.2. Sample Pretreatment

Tissues were carefully pulled out from the frozen tubes, cut into pieces, weighed about 50 mg, added 10 times extraction buffer, and extracted protein with a grinding mill. The extracted samples were spun to remove the residue of tissues. The protein concentration of the extracted sample solution was measured with a BCA Protein Assay kit (Pierce). Proteins extracted from tissue were precipitated with a TCA Protein Precipitation Kit (QYBIO). The protein pellet was dried out by SpeedVac. The pellet was subsequently dissolved with 8 M urea in 100 mM Tris-Cl (pH 8.5, Sigma). Tris(2-carboxyethyl)phosphine (TCEP, final concentration is 5 mM, Thermo Scientific) and iodoacetamide (final concentration is 10 mM, Sigma) were added to the solution and incubated at room temperature for 20 and 15 minutes for reduction and alkylation, respectively. The solution was digested with LysC at 1 : 100 (*w*/*w*) (Promega) for 4 h and then diluted four times and digested with trypsin at 1 : 50 (*w*/*w*) (Promega) for 16 h. The digested peptide mixtures were labelled with the TMT kit (6-plex, Thermo).

#### 2.1.3. Liquid Chromatography Tandem Mass Spectrometry Analysis of Peptides

For multidimensional protein identification technology (MudPIT), total peptide mixtures were pressure loaded onto a biphasic-fused silica capillary column. The entire column setting (biphasic column-union-analytical column) was placed in line with an Agilent 1200 quaternary high-performance liquid chromatography pump (Palo Alto, CA) for MS analysis. The digested proteins were analyzed using a 12-step MudPIT separation method [21] as described previously.

#### 2.1.4. Mass Spectrometry Condition

Data-dependent tandem mass spectrometry (MS/MS) analysis was performed with an Orbitrap mass spectrometer (Thermo Scientific, San Jose, CA). Peptides eluted from the liquid chromatography system were directly electrosprayed into the mass spectrometer with a distal 1.5 kV spray voltage. One acquisition cycle included one full-scan MS spectrum (*m*/*z*, 300–1800), followed by the top 20 MS/MS events.

#### 2.1.5. Data Analysis

The acquired MS/MS data were analyzed against a Uniprot Homo sapiens database (database released on Sep. 24, 2015) using Integrated Proteomics Pipeline (IP2, http://integratedproteomics.com/). To estimate peptide probabilities and false discovery rates accurately, we used a decoy database containing the reversed sequences of all the proteins appended to the target database. Carbamidomethylation of cysteine was considered as a static modification.

### 2.2. Immunohistochemistry

#### 2.2.1. Sample

The aortic arch tissue's paraffin sections of another 20 patients with AAD and 10 healthy donors were performed and provided by the Pathological Sample Library of Zhongshan Hospital of Fudan University. The standardized method to fix the tissues would be operated by professional staff as follows: approximately 1.0 × 1.0 × 0.2 cm^3^ of tissue would be extracted during surgery and fixed with formalin rapidly for 24 h; paraffin sections are performed and stored in the sample library. The basic clinical information is shown in Supplementary Table 3. The study was also approved by the ethics committee of Zhongshan Hospital of Fudan University.

#### 2.2.2. Immunohistochemistry Method

The expression of integrin alpha 3 (ITGA-3) and integrin alpha 5 (ITGA-5) were compared stained with DAB staining and hematoxylin. Briefly, paraffin sections were dewaxed, and antigen was retrieved by citric acid buffer. The primary antibody of ITGA-3 and ITGA-5 is the rabbit antibody (Abcam, ab131055 and ab239400, respectively, 1 : 100). The corresponding secondary antibody is HRP-goat anti-rabbit IgG (H+L) (GXYbio, S8002, 1 : 100). The sections were reacted with the DAB kit (ZSGB-BIO, China, ZLI-9019). Eight separate views (magnification = original × 400) were randomly selected. The integrated option density (IOD) of ITGA-3 and ITGA-5 was chosen to determine the protein semiquantitative expression. Free ImageJ software (version 1.2; WS Rasband, National Institute of Health, Bethesda, MD) was used to conduct deconvolution and downstream analysis [22], and then, the Mann-Whitney test of the IOD was performed using SPSS software (version 25.0, IBM).

## 3. Results

### 3.1. Identification Differential Abundance Proteins (DAPs) of Ascending Aorta Tissues in Patients with AAD Using TMT Quantitative Proteomics

**Figure 2:**
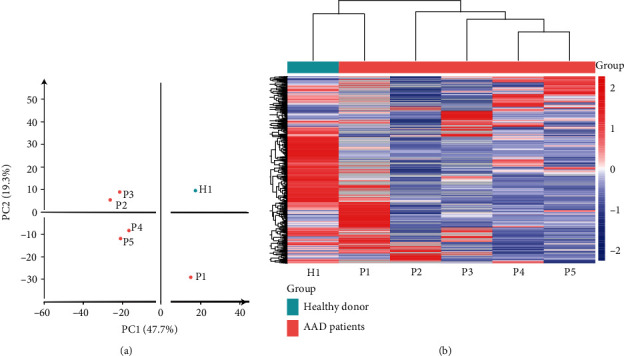
Principal component analysis (PCA) and heat map of proteome data. (a) Principal component analysis of the proteome data in a 2D graph of PC1 and PC2. (b) Expression heat map of the proteome data in the samples.

TMT-labelled MS analysis was performed on fresh aortic arch tissues of 5 AAD patients and one healthy donor. Principal component analysis (PCA) clustergram was created using OmicsBean software from proteins identified by quantitative MS. Except patient 1, other patients have consistent clustering as shown in PCA and heat map analysis (Figure 2). Patient 1 might fall in different subtypes of AAD compared with the other patients.

A total of 809 proteins were identified from individual unique peptides (Supplementary Table 2). Limited by the number of the healthy control, we defined the identified proteins as DAPs if there was a log_2_FC in excess of 2 or in less of -2.132 proteins changed significantly (32 upregulated and 100 downregulated) in AAD patients compared with the healthy donor (Tables 1 and 2).

**Figure 3:**
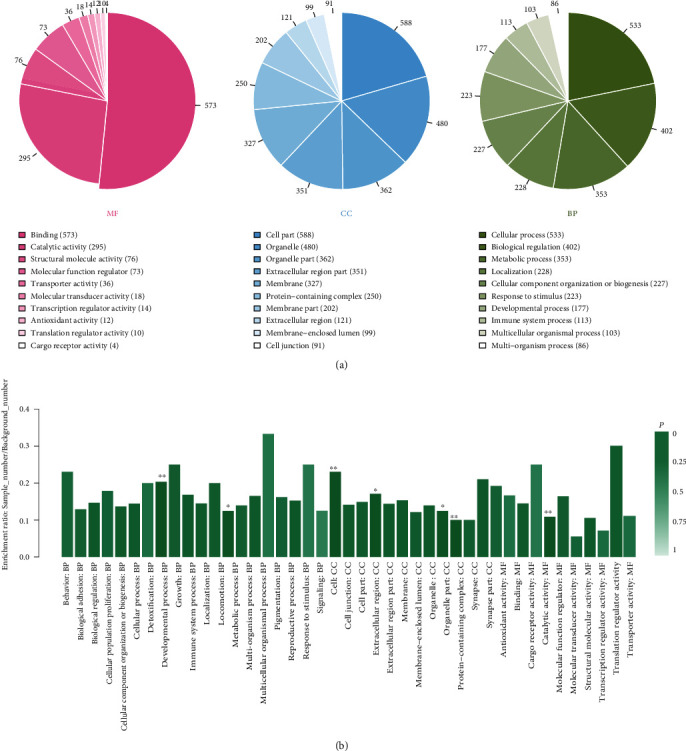
The Gene Ontology (GO) enrichment of 132 differential abundance proteins (DAPs). (a) Sorted by descending order of the number of protein associated with the listed GO ID. (b) Sorted by descending order of *P* value for the GO enrichment terms. Because of the limited number of patients, we considered DAPs as meaningful only as strong differently expressed (∣log_2_FC | >2).

**Figure 4:**
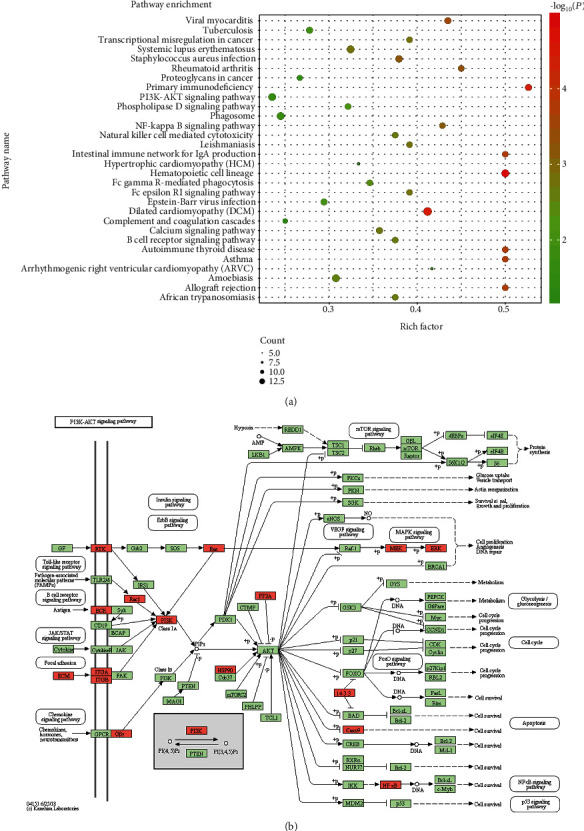
Kyoto Encyclopedia of Genes and Genomes (KEGG) pathway enrichment analysis for 132 DAPs between health donor vs. patients. (a) Top 30 significantly enriched pathways were shown in the senior bubble chart. The rich factor is the ratio of DAP numbers annotated in this pathway term to all gene numbers annotated in this pathway term (*P* < 0.01). (b) Most of downregulated DAPs (*n* = 16/51) were found in the PI3K-AKT signaling pathway.

**Figure 5:**
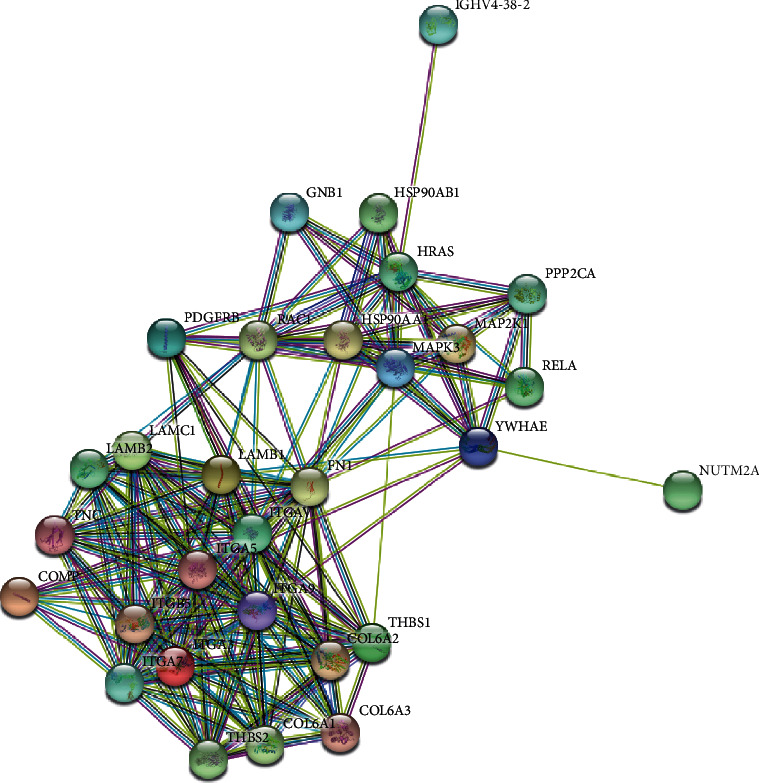
The protein-protein interaction networks is analyzed by string software. Different line colours represent the types of evidence used in predicting the associations: gene fusion (red), neighborhood (green), cooccurrence across genomes (blue), coexpression (black), experimental (purple), association in curated databases (light blue), or comentioned in PubMed abstracts (yellow).

To explain the cellular localization and associated functions of DAPs, gene Ontology analysis (GO analysis) was performed by Blast2GO software (version 4) to provide further understanding of these results by biological processes, cell components, and molecular function (Figure 3). According to the GO results, most DAPs were located in the extracellular region part and participated in several functions, such as cell junction, metabolic process, and developmental process, indicating that proteins involved in extracellular activities were important in AAD tissues. Kyoto Encyclopedia of Genes and Genomes (KEGG) pathway enrichment analysis was used to classify the functional annotations of DAPs. Interestingly, among the 30 significantly enriched pathways, most of downregulated DAPs (*n* = 16/51) were found in the PI3K-AKT signaling pathway (Figure 4). Protein-protein interaction (PPI) analysis further suggested that ITGA-3 and ITGA-5 were important nodes in the PI3K-AKT signaling pathway (Figure 5).

### 3.2. Verification of the Levels of ITGA-3 and ITGA-5 by Immunohistochemistry

**Figure 6:**
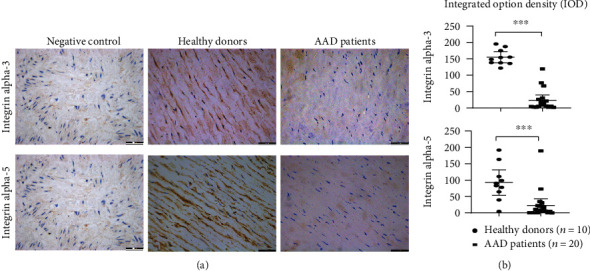
The biomarkers (ITGA-3, ITGA-5) verified by immunohistochemistry (IHC). (a) The black arrows present positive signals of ITGA-3 and ITGA-5 staining by DAB in healthy donors. Negative control: the section was stained with secondary antibodies only. Scale bars = 200 *μ*m. (b) The IODs of ITGA-3 and ITGA-5 in AAD patients were significantly lower compared with healthy donors (^∗∗∗^*P* < 0.001).

The levels of ITGA-3 and ITGA-5 in pathological sections of 20 AAD patients and 10 healthy donors were further assessed by immunohistochemistry (Figure 6(a)). The basic information of them is shown in Supplementary Table 3. The IOD of ITGA-3 and ITGA-5 in AAD patients was significantly lower compared with healthy donors (*P* < 0.001) (Figure 6(b)).

## 4. Discussion

AAD of the ascending aorta is a life-threatening cardiovascular emergency with a mortality rate of 1% to 2% per hour early after the symptom onset [1, 3]. With increasing incidence of hypertension, the morbidity of AAD has increased annually in recent years [23]. However, the detailed molecular mechanisms of this disease have yet to be clarified. Finding out important proteins in dissected aorta tissues of patients with AAD can help to elucidate the pathogenesis of this disease.

In this study, the quantitative proteomics showed that 100 proteins were significantly downregulated while 32 upregulated in dissected aorta tissues of AAD patients compared with those in ascending aorta tissue of the healthy donor. Bioinformatic analysis revealed that most DAPs were primarily located in the extracellular region part and their biological functions mainly focused on cell junction, metabolic process, and developmental process. Interestingly, the PI3K-AKT signaling pathway was selected by KEGG analysis, which has been reported involved in a series of biological processes by transducing stimulatory extracellular signals to the nucleus, including proliferation, apoptosis, angiogenesis, and tumor growth [24, 25]. Liu and his colleagues demonstrated that the PI3K/AKT signaling transduction pathway was involved in rat vascular smooth muscle cell proliferation induced by apelin-13 [18]. Other studies also have shown that the 0PI3K/AKT signaling pathway contributes to VSMC dysfunction, vasoconstriction, and vascular remodeling [19, 26]. However, few studies reported the molecule or protein in the PI3K/AKT signaling pathway contributing to the VSMC dysfunction in human aorta tissues.

In this study, we found that integrins, especially ITGA-3 and ITGA-5, were significantly downregulated in dissected aorta tissues of AAD patients. As expected, ITGA-3 and ITGA-5 showed a strong downexpression in the cytomembrane and cytoplasm. As a kind of cell surface adhesion molecule with signal transduction function, integrins are key transmembrane protein upstream in the P13K-AKT signaling pathway. By connecting the extracellular matrix with actin cytoskeleton, it can maintain cell morphology and mediate cell adhesion, proliferation, differentiation, and other physiological processes according to the types of extracellular ligands. Downregulation of integrin may affect the stability of cytoskeleton, thus ultimately affecting the physiological function of cells [27, 28]. Taken together, downregulation of ITGA-3 and ITGA-5 might be involved in the pathogenesis of AAD by reducing the adhesion between cell and extracellular matrix and modulating the focal adhesion pathway.

The proteomic approach provides an exciting platform for determining the pathogenesis of aortic dissection. As an initial step, our study identified the downregulation of ITGA-3 and ITGA-5, both of which may participate in AAD pathogenesis via the focal adhesion pathway. To make up for the control sample shortage in the proteomic study, we obtained the pathological sections of another 10 healthy donors and 20 AAD patients from the Pathological Sample Library of Zhongshan Hospital to further make our results credible in the following validation test. The performance of the PI3K-AKT signaling pathway needs to activate downstream proteins, which provides insights into the specific molecule or signaling pathway contributing to the pathogenesis of aortic dissection. It needs to be further explored in animals and humans.

## 5. Conclusions

The aortic arch tissues of patients with AAD show a large number of DAPs, the molecular functions of which were primarily cell junction. In particular, ITGA-3 and ITGA-5 were highly differentially expressed in these tissues. Downregulation of the two proteins may contribute to the progression of AAD, which may serve as a diagnostic biomarker and a novel therapeutic target in AAD.

## Figures and Tables

**Table 1:** Upregulated differential abundance proteins in AAD patients (*n* = 32).

Accession	Expression of healthy donor	Mean expression of AAD patients (*n* = 5)	log_2_FC	Description
Q14315	324.67	7870.896	4.599	Filamin-C
Q5U0D2	204	3628.784	4.153	Transgelin
P37802	2044.82	28287.816	3.79	Transgelin-2
B0AZV6	471.5	5283.69	3.486	cDNA, FLJ79546, highly similar to SH3 domain-binding glutamic acid-rich-like protein
J3KND3	180	1960.68	3.445	Myosin light polypeptide 6
A8KAH9	565	6078.1	3.427	RAP1A, member of RAS oncogene family
A0A024QZQ2	267	2674.5	3.324	Prosaposin (Variant Gaucher disease and variant metachromatic leukodystrophy), isoform CRA_b
A0A024RAB6	368.77	3658.2	3.31	Heparan sulfate proteoglycan 2 (Perlecan), isoform CRA_b
P08294	718	6862.38	3.257	Extracellular superoxide dismutase [Cu-Zn]
F2RM37	315.5	2933.168	3.217	Coagulation factor IX
A0A024R321	236.44	2162.786	3.193	Filamin-B, beta (actin binding protein 278), isoform CRA_a
B4DHX4	241.5	2181.672	3.175	cDNA FLJ52902, highly similar to Rab GDP dissociation inhibitor alpha
A6NIZ1	565	5073.772	3.167	Ras-related protein Rap-1b-like protein
A0A024QZX3	180	1546.584	3.103	Serpin peptidase inhibitor, clade B (Ovalbumin), member 6, isoform CRA_a
Q53HQ0	270	2227.7	3.045	Flotillin 1 variant (fragment)
A6NLG9	252.8	2077.26	3.039	cDNA FLJ36740 fis, clone UTERU2013322, highly similar to Biglycan
A5YM53	229	1812.1875	2.984	ITGAV protein
V9HWF6	287.5	2256.258	2.972	Alpha 1-acid glycoprotein
B2RDY9	190	1426.9175	2.909	Adenylyl cyclase-associated protein
B4DLV7	311.17	2185.602	2.812	cDNA FLJ60299, highly similar to Rab GDP dissociation inhibitor beta
P01031	461	3067.166667	2.734	Complement C5
Q9NZM1	201	1334.328	2.731	Myoferlin
P13645	870.5	5446.79	2.645	Keratin, type I cytoskeletal 10
P08779	1462.67	8870.308	2.6	Keratin, type I cytoskeletal 16
B1N7B8	201	1028.0625	2.355	Cryocrystalglobulin CC1 kappa light chain variable region (fragment)
A0A024RBX9	256	1281.3	2.323	Pyruvate dehydrogenase E1 component subunit alpha
Q7Z7J6	241.25	1205.772	2.321	Actin alpha 1 skeletal muscle protein
B7Z6P1	266.05	1306.056	2.295	cDNA FLJ53662, highly similar to actin, alpha skeletal muscle
A8K4W0	347	1656.5	2.255	40S ribosomal protein S3a
A0A024R821	451	1989.75	2.141	Eukaryotic translation initiation factor 3 subunit B
P07357	947	4078	2.106	Complement component C8 alpha chain
G3V4G1	274	1140	2.057	Neuroguidin (fragment)

Note: based on protein abundance. A protein was defined as DAPs if there was a log2FC in excess of 2. FC: fold change.

**Table 2:** Downregulated DAPs in AAD patients (*n* = 100).

Accession	Expression of healthy donor	Mean expression of AAD patients (*n* = 5)	log_2_FC	Description
H3BQZ7	2286.5	69.33333333	-5.04344799	HCG2044799
Q6GMX6	24204.11	1024.248	-4.56261507	IGH@ protein
Q6MZQ6	21830.2	939.36	-4.53850338	Putative uncharacterized protein DKFZp686G11190
Q6N089	19877.09	909.564	-4.4497876	Putative uncharacterized protein DKFZp686P15220
V9HW68	19889.09	1004.376	-4.3076059	Epididymis luminal protein 214
Q92626	2750	187.25	-3.87639399	Peroxidasin homolog
A0A024R971	5098	368.058	-3.79192634	Fibromodulin, isoform CRA_a
A0A087X2C0	5633	410.31	-3.77911725	Ig mu chain C region
A0A024R9G4	1679	122.6666667	-3.77478706	Family with sequence similarity 49, member B, isoform CRA_a
A0A0G2JPD4	6460.29	505.912	-3.67464056	Uncharacterized protein
B0YJ88	4307	350.696	-3.61839044	Radixin
Q96C32	9160.43	752.5633333	-3.60553042	Polyubiquitin-C
A8K3Q7	19376	1632.302	-3.56929086	Annexin
P43652	2496.5	211.875	-3.55862163	Afamin
A0A024R0S6	5116.29	460.624	-3.47343655	EH-domain containing 2, isoform CRA_a
Q9NZU5	5750	529.346	-3.44127902	LIM and cysteine-rich domains protein 1
F2ZC06	3779.75	349.452	-3.43512461	Thyroid hormone receptor interacting protein 6 isoform 1
P26006	4296	414.25	-3.37442039	Integrin alpha 3/beta 1
A8MX94	5754.4	581.104	-3.30779722	Glutathione S-transferase P
B2R6V9	1398	142	-3.29940153	cDNA, FLJ93141, highly similar to Homo sapiens coagulation factor XIII, A1 polypeptide (F13A1), mRNA
P01023	3824.29	397.764	-3.26520731	Alpha 2-macroglobulin
O94832	1636	170.944	-3.25857706	Unconventional myosin-Id
B4DKT9	4013	422.1666667	-3.24879658	cDNA FLJ54052, highly similar to alpha 1 catenin (cadherin-associated protein)
D6RA82	5307.67	564.375	-3.23335269	Annexin
B4DQG5	2126.25	227.9175	-3.22172763	cDNA FLJ54122, highly similar to Cytosol aminopeptidase (EC 3.4.11.1)
P12235	1117	125.3333333	-3.15578712	ADP/ATP translocase 1
A0A024QZV0	1431	160.625	-3.15525531	HCG1811539, isoform CRA_b
Q6UVK1	1875	210.564	-3.15455989	Chondroitin sulfate proteoglycan 4
A0A0B4J1R6	1682.75	191.546	-3.13505805	Transketolase
B4DRV4	2956	340.5	-3.11791957	cDNA FLJ55667, highly similar to secreted protein acidic and rich in cysteine
A0A024RC65	4177	531.732	-2.97369595	HCG1991735, isoform CRA_a
A0A024RDE1	1252	161.95	-2.95061419	SPARC-like 1 (Mast9, hevin), isoform CRA_a
Q8TCD0	14216	1858.354	-2.93541833	Uncharacterized protein
A0A024RAC9	253	34	-2.89553073	Zinc finger, UBR1 type 1, isoform CRA_c
O76024	2314	313	-2.8861543	Wolframin
B4DQX8	3990	547.875	-2.86447007	cDNA FLJ51723, highly similar to DCC-interacting protein 13 alpha (fragment)
V9HWK1	2754	387.9425	-2.82761382	Triosephosphate isomerase
P49327	1231.5	174.842	-2.81629294	Fatty acid synthase
B4DNM8	360	51.75	-2.79836614	cDNA FLJ53395, highly similar to Prolyl 3-hydroxylase 1 (EC 1.14.11.7)
A0A087WZW8	14216	2052.316	-2.7921908	Protein IGKV3-11
A0A087WUS7	2422	354.7	-2.77152763	Ig delta chain C region
B4E3A8	1457	216.3333333	-2.75167299	cDNA FLJ53963, highly similar to leukocyte elastase inhibitor
A8K3B6	406	60.5	-2.74647268	Nonspecific protein-tyrosine kinase
S6C4R6	12602.88	1884.586	-2.74143392	IgG L chain
A0A087WYL9	14216	2132.92	-2.73661383	Ig kappa chain C region
Q6GMX0	11199.11	1698.368	-2.72116309	Uncharacterized protein
Q6PJF2	9283.64	1452.93	-2.67572538	IGK@ protein
P27824	1292.5	204.5	-2.65999153	Calnexin
Q6P5S8	14216	2252.75	-2.65775646	IGK@ protein
A0A087X130	12702.62	2022.842	-2.65067055	Ig kappa chain C region
A0A087WWT3	646	104	-2.63495064	Serum albumin
V9HW34	9283.64	1502.036	-2.62777119	Epididymis luminal protein 213
Q8NCL6	2879.25	479.72	-2.58542857	cDNA FLJ90170 fis, clone MAMMA1000370, highly similar to Ig alpha 1 chain C region
Q9NPP6	3076.09	515.486	-2.57709256	Immunoglobulin heavy chain variant (fragment)
Q96K68	2872.08	483.564	-2.57031719	cDNA FLJ14473 fis, clone MAMMA1001080, highly similar to Homo sapiens SNC73 protein (SNC73) mRNA
B4DEA3	218	37	-2.55873096	cDNA FLJ56531, highly similar to UV excision repair protein RAD23 homolog B
Q96CD0	228.5	41	-2.47849835	F-box/LRR-repeat protein 8
V9HWF2	1684.5	306.8175	-2.45686775	Malate dehydrogenase
A8K4C8	5080	925.6666667	-2.45626382	60S ribosomal protein L13
A0A024RDY2	860	158.994	-2.43536434	Tumor protein, translationally controlled 1, isoform CRA_a
Q6MZV6	2675.38	500.17	-2.41925339	Putative uncharacterized protein DKFZp686L19235
A0A024RB01	2564	484.4	-2.40412549	Integrin, alpha 5 (fibronectin receptor, alpha polypeptide), isoform CRA_b
A6NLN1	652	123.9175	-2.39549202	Polypyrimidine tract binding protein 1, isoform CRA_b
P07204	989	190.33	-2.37746754	Thrombomodulin
F6U211	253	49.46666667	-2.35460879	40S ribosomal protein S10
P63267	1432	283.6666667	-2.33576296	Actin, gamma-enteric smooth muscle
A0A0C4DFX3	829	164.6666667	-2.33182356	EMILIN-1
A6NNI4	571	114.3333333	-2.32024467	Tetraspanin
A0A024RA21	843	170.8	-2.30322466	Secernin 1, isoform CRA_a
B2R5H0	1174	245.915	-2.25520077	Protein S100
B4DPU3	7412	1556.5	-2.2515573	cDNA FLJ56548, highly similar to elongation factor 2
Q6MZU6	6876.26	1445.532	-2.25002355	Putative uncharacterized protein DKFZp686C15213
A0A024R1N1	1066	224.5	-2.24742009	Myosin, heavy polypeptide 9, nonmuscle, isoform CRA_a
A0A075B6N8	6872.24	1450.324	-2.24440519	Ig gamma-3 chain C region (fragment)
B7Z539	898	192.05	-2.22523348	cDNA FLJ56954, highly similar to interalpha trypsin inhibitor heavy chain H1
A0A024R930	232	49.75	-2.22135637	Proteoglycan 4, isoform CRA_a
A8K9M5	1651.17	354.448	-2.21984277	cDNA FLJ77947, highly similar to human complement protein C8 beta subunit mRNA
A0A024R7Z5	412.5	89.2075	-2.20915721	Syndecan binding protein (Syntenin), isoform CRA_c
B4DDS8	846	184.5	-2.19703685	cDNA FLJ56686, moderately similar to FADD protein
A0A024R3W7	239	52.5	-2.18662129	Eukaryotic translation elongation factor 1 beta 2, isoform CRA_a
A0A087WUN8	371	82	-2.17772337	Syntaxin-binding protein 2
B4DTX5	1412	314.816	-2.16515932	cDNA FLJ60072, highly similar to Homo sapiens sorbin and SH3 domain containing 1 (SORBS1), transcript variant 6, mRNA
Q9BVK6	267	59.75	-2.15982912	Transmembrane emp24 domain-containing protein 9
Q9Y217	430	96.33333333	-2.15822967	Myotubularin-related protein 6
Q8IWB1	194	43.5	-2.15696935	Inositol 1,4,5-trisphosphate receptor-interacting protein
A0A075B6G3	818.5	183.5766667	-2.15659972	Dystrophin
K7EQQ3	6367	1439.5	-2.14504598	Keratin, type I cytoskeletal 9
B4E2F9	1999.32	455.736	-2.13323916	cDNA FLJ57038, highly similar to Filamin-A
C9JAK5	1520	346.7	-2.13231158	ADP-ribosylation factor 4
B7Z9B7	1412	322.75	-2.12925109	cDNA FLJ54732, moderately similar to sorbin and SH3 domain-containing protein 1
B2ZZ89	884	202.512	-2.12603897	Epididymis luminal protein 102
A0M8W4	902	207.5	-2.1200161	Ubiquitin-conjugating enzyme E2 variant 2
B0QZ18	3015	723.296	-2.05949992	Copine-1
A0A024RAX0	676	162.25	-2.05880477	Matrix Gla protein
B0QY01	225	54.125	-2.05555798	Target of Myb protein 1 (fragment)
A0A087WU08	2574	619.875	-2.05396283	Haptoglobin
A0A024R6P0	2748	666.416	-2.04388706	Serpin peptidase inhibitor, clade A (alpha 1 antiproteinase, antitrypsin), member 3, isoform CRA_c
A0A024RD41	3012	731.6666667	-2.04146333	RAB23, member RAS oncogene family, isoform CRA_a
A0A024R1Z6	242.5	59	-2.03919789	Vesicle amine transport protein 1 homolog (T. californica), isoform CRA_a
Q59EQ1	478	119.5	-2	Cadherin 11, type 2 isoform 1 preproprotein variant (fragment)

Note: based on protein abundance. A protein was defined as DAPs if there was a log2FC in less of -2.

## Data Availability

The datasets used and/or analyzed during the current study are available from the corresponding author on reasonable request.

## Abbreviations

AAD: Acute aortic dissection
DAPs: Differential abundance proteins
TMT: Tandem mass tag
ITGA-3: Integrin alpha 3
ITGA-5: Integrin alpha 5
MMPs: Matrix metalloproteinase
VSMC: Vascular smooth muscle cell
MS: Mass spectrometry
CTA: Computed tomography angiography
TCEP: Tris(2-carboxyethyl)phosphine
MudPIT: Multidimensional protein identification technology
IOD: Integrated option density
PCA: Principal component analysis
GO analysis: Gene Ontology analysis
KEGG: Kyoto Encyclopedia of Genes and Genomes
PPI analysis: Protein-protein interaction analysis.

## Supplementary Materials

Supplementary 1 Supplementary Table 1 Basic data between AAD patients and healthy control in the TMT method. Note: ^a^“0” is no; “1” is yes. ^b^According to the more popular Stanford system, dissections involving the ascending aorta are classified as type A, whereas those involving only the descending aorta are classified as type B.

Supplementary 2 Supplementary Table 2 List of expreesion of all identified proteins between the two groups in the TMT method.

Supplementary 3 Supplementary Table 3 Basic data between AAD patients and healthy controls in immunohistochemistry. Note: ^a^“0” is no; “1” is yes. ^b^According to the more popular Stanford system, dissections involving the ascending aorta are classified as type A, whereas those involving only the descending aorta are classified as type B.
